# P2Y_2_ Receptor Controls TLR3‐Induced Chemokine Production in Intestinal Epithelial Cells

**DOI:** 10.1155/jimr/4814045

**Published:** 2026-08-30

**Authors:** Abdoul Karim Ouattara, Fariborz Bahrami, Radu Adrian Turcitu, Filip Kukulski, Joanna Lecka, Julie Pelletier, Nicolly Espindola Gelsleichter, Tais Vidal, Jean Sévigny

**Affiliations:** ^1^ Département de Microbiologie-Infectiologie et d’immunologie, Centres PROTEO et ARThrite, Faculté de Médecine, Université Laval, Québec City G1V 0A6, Quebec, Canada, ulaval.ca; ^2^ Axe Maladies Infectieuses et Immunitaires, Centre de Recherche du CHU de Québec - Université Laval, Québec City G1V 4G2, Quebec, Canada, ulaval.ca

**Keywords:** CXCL10/IP-10, CXCL8/IL-8, intestinal epithelial cells, *P2RY2*, TLR3

## Abstract

Toll‐like receptors (TLRs) and P2 receptors are key regulators of innate immunity. During infection, pathogen‐associated molecular patterns (PAMPs) activate TLRs, whereas extracellular nucleotides engage P2 receptors and influence inflammatory responses. Here, we show that polyinosinic:polycytidylic acid (poly[I:C])‐activated TLR3 and P2Y_2_ signalling functionally interact in intestinal epithelial cells (IECs) to regulate chemokine production. HT‐29 cells were stimulated with PAMPs, including the TLR3 agonist poly(I:C), in the presence or absence of P2 receptor signalling inhibitors. CXCL8/IL‐8 or CXCL10/IP‐10 secretion was assessed by ELISA and mRNA expression by quantitative real‐time PCR (RT‐qPCR). Primary IECs from P2Y_2_ knock‐out (KO) and wild‐type (WT) mice were also treated with poly(I:C), and CXCL1/KC secretion was measured. Poly(I:C) stimulation induced robust CXCL8/IL‐8 and CXCL10/IP‐10 release in HT‐29 cells. This response was inhibited by nucleotide scavenging, P2 receptor blockade, and P2Y_2_ targeting using either specific antagonists or siRNAs. P2Y_2_ was the dominant receptor expressed, and its ligands ATP/UTP constitutively released by these cells amplified CXCL8/IL‐8 production induced by a suboptimal concentration of poly(I:C), while alone they had no effect. In support of these findings, primary IECs from P2Y_2_ KO mice secreted significantly less CXCL1/KC than WT controls. Altogether, extracellular nucleotide signalling regulates TLR3‐induced chemokine release in IECs through P2Y_2_ receptors.

## 1. Introduction

The intestinal epithelium plays a critical role not only in nutrient absorption and barrier maintenance but also in coordinating immune responses to microbial and viral stimuli [1, 2]. Among the various pattern recognition receptors expressed by intestinal epithelial cells (IECs), toll‐like receptor 3 (TLR3) recognises viral or host‐derived double‐stranded RNA as well as the synthetic analogue polyinosinic:polycytidylic acid (poly[I:C]) [3, 4]. TLR3 activation triggers innate immune responses, including the production of type I interferons and pro‐inflammatory chemokines like CXCL8 (IL‐8) [5] and CXCL10 (IP‐10) [6]. These chemokines recruit neutrophils [7] and T cells [8], respectively, and are elevated in the inflamed mucosa of patients with inflammatory bowel disease [9, 10].

Emerging evidence suggests that extracellular nucleotides (e.g., ATP, ADP, UTP, UDP and UDP‐sugar), acting through specific nucleotide receptors ubiquitously expressed on the cell surface, play a key role in modulating immune responses [7, 11–14]. Inflammation has been shown to upregulate the expression of both P2Y_2_ and P2Y_6_ receptors in the colonic mucosa of colitis mice [15]. As previously demonstrated by our research team, the P2Y_6_ receptor (activated by UDP) is expressed by both mouse and human IECs and has been implicated in enhancing cytokine signalling and promoting recruitment of immune cells like T cells and macrophages [8, 16]. The P2Y_2_ receptor, activated by ATP and UTP, modulates key functions in IECs, including leukocyte recruitment via NF‐κB‐dependent ICAM‐1 upregulation [17], epithelial ion transport through PLC/IP3/Ca^2+^‐mediated bicarbonate secretion [18], and wound healing by promoting cell migration and microtubule stabilisation via PI3K/Akt–dependent GSK3β inhibition [19].

In vivo, P2Y_2_ receptor activation enhances mucosal repair and reduces bacterial translocation in colitis, underscoring its therapeutic relevance in intestinal inflammation [19]. Finally, a recent study reported that the *P2RY2* gene is significantly overexpressed in HT‐29 colorectal cancer (CRC) cells compared to HCEC‐1CT, a normal‐like colonic epithelial cell line [20].

In line with the findings of pioneering studies conducted by our research team, which demonstrated that TLR2‐ or TLR4‐mediated cytokine production is regulated by P2 receptors in monocytes and neutrophils [7, 11, 12], we hypothesised that functional interaction between TLR3 and the extracellular nucleotide signalling pathway is also required for chemokine production. This crosstalk warrants investigation for several reasons. First, both TLR3 and P2 receptors are activated during tissue damage and infection—TLR3 by viral or host‐derived RNA and P2 receptors by nucleotide released from stressed or dying cells [21, 22]. Second, their signalling pathways converge on common transcription factors (e.g., NF‐κB, AP‐1) and calcium‐dependent effectors, raising the possibility of synergistic or antagonistic interactions [23, 24]. Third, understanding whether and how these pathways integrate could reveal new mechanisms by which the intestinal epithelium discriminates between homeostatic and inflammatory conditions and may identify novel therapeutic targets for inflammatory bowel disease or viral enteropathies.

In this study, we provide evidence for a functional interaction between TLR3 and P2Y_2_ receptors in IECs, demonstrating that nucleotide signalling potentiates chemokine secretion in response to the viral mimic poly(I:C). Our data elucidate how extracellular nucleotides regulate chemokine expression during viral‐like inflammation and suggest that targeting the P2Y_2_ receptor could modulate pathological chemokine production in the gut.

## 2. Materials and Methods

### 2.1. Materials

DMEM/F12, L‐glutamine, fetal bovine serum (FBS), Hank’s balanced salt solution (HBSS) containing Ca^2+^ and Mg^2+^, HEPES, and antibiotic–antimycotic solution were obtained from Wisent (St‐Bruno, QC, Canada). Advanced DMEM/F12, the PureLink Genomic DNA Mini Kit, Quant‐iT RNA BR Assay Kit, TRIzol reagent, RNase‐free DNase I (AM2222), SuperScript III reverse transcriptase, RNaseOUT recombinant ribonuclease inhibitor, dNTPs, DTT, optical 384‐well reaction plates, custom‐made primers, and the 1 kb Plus DNA Ladder were purchased from Invitrogen (Burlington, ON, Canada).

Human CXCL8/IL‐8 and CXCL10/IP‐10 and mouse CXCL1/KC DuoSet ELISA kits were purchased from R&D Systems, Inc. (Minneapolis, MN, USA). Normocin, Pam3CSK4, poly(I:C), *Salmonella typhimurium* flagellin, Gardiquimod, and 2‐aminopurine were obtained from InvivoGen (San Diego, CA, USA). The 2‐aminopurine solution was prepared in phosphate‐buffered saline and glacial acetic acid at a ratio of 200:1.

Lipopolysaccharide from *Escherichia coli* O111:B4, random nonamers (R7647), Grade VII potato apyrase, ATP, ADP, UTP, UDP, adenosine, adenosine 5‐(γ‐thio)‐triphosphate (ATP‐γ‐S), suramin, cycloheximide, actinomycin D, endotoxin‐free saline, and endotoxin‐free water were purchased from Sigma–Aldrich (Oakville, ON, Canada). A 5 mg/mL LPS stock solution was prepared in endotoxin‐free saline, dispersed for 10 min in a sonication water bath, and diluted in the culture medium immediately before stimulation.

Bafilomycin A1 was obtained from AG Scientific (San Diego, CA, USA). MRS2500, AR‐C118925XX, NF157, and 5‐BDBD were obtained from Tocris Bioscience (Minneapolis, MN, USA), whereas Reactive Blue 2 (RB‐2) was purchased from ICN Biochemicals Inc. (Aurora, OH, USA). Stock solutions of suramin, RB‐2, MRS2500, and NF157 were prepared at 10 mM in endotoxin‐free water, whereas AR‐C118925XX and 5‐BDBD were dissolved at 10 mM in DMSO. All solutions were sterilised by filtration.

Three siRNA duplexes targeting human *P2RY2* were used, together with a non‐targeting control siRNA (Catalogue No. SR303327) was purchased from OriGene Technologies Inc. (Rockville, MD, USA). Millex‐GP 0.22‐µm syringe‐driven filter units and Immobilon‐P membranes were obtained from Millipore (Billerica, MA, USA). The RNeasy Mini Kit and QuantiTect Reverse Transcription Kit were purchased from Qiagen (Mississauga, ON, Canada). Taq DNA polymerase was obtained from New England Biolabs (Ipswich, MA, USA), and FastStart SYBR Green Master Mix was purchased from Roche (Mannheim, Germany).

### 2.2. Cell Culture and Treatment

The human colon adenocarcinoma cell line HT‐29 (female, colon; ATCC HTB‐38, RRID: CVCL_0320) was purchased from the American Type Culture Collection (ATCC, Manassas, VA, USA) in 2009 and maintained as monolayers in DMEM/F‐12 growth medium supplemented with 2 mM L‐glutamine, 1X antibiotic–antimycotic solution, 25 mM Hepes, 100 μg/mL Normocin (anti‐mycoplasma reagent), and 10% heat‐inactivated foetal bovine serum at 37°C in a 95% air/5% CO_2_ atmosphere. Cell line identity was authenticated by ATCC using cytochrome oxidase I (COI) species verification and short tandem repeat (STR) profiling, which showed a 100% match to the HT‐29 reference profile. This cell line is not listed as misidentified or contaminated according to the International Cell Line Authentication Committee database. Cells were regularly monitored for the presence of *Mycoplasma* spp. by means of a conventional PCR test [25] using 5 μg of extracted genomic DNA (PureLink genomic DNA mini kit) as a template. Unless otherwise indicated, the cells from passages 2–3 were seeded (2 × 10^5^/well) in 24‐well plates containing 1 mL of medium for 24 h prior to treatment.

To mimic in vivo mucosal inflammatory conditions, in some experiments, cells were primed with pro‐inflammatory cytokines. Specifically, cells were seeded at a density of 2 × 10^6^/well in 24‐well plates and, on the following day, were incubated in a serum‐free medium for 24 h. They were then treated with fresh serum‐free medium containing recombinant human TNF and IFN‐γ (each at 1 ng/mL) for 6 h to induce an inflammatory‐like stress condition [15, 26]. For cell counting and subculturing, cells were dispersed with a 0.25 % trypsin solution. Cell viability consistently exceeded 95%, as determined by the trypan blue dye exclusion assay.

### 2.3. CXL8/IL‐8 and CXCL10/IP‐10 Production Assays

HT‐29 cells in 24‐well plates were stimulated with pathogen‐associated molecular patterns (PAMPs), namely, Pam_3_CSK_4_ (TLR1/2 agonist), poly(I:C) (TLR3), flagellin (TLR5), and gardiquimod (TLR7/8), all at 1 μg/mL, or 0.1 μg/mL LPS, in the absence or presence of the following inhibitors of extracellular nucleotide signalling: apyrase (0.5 units/mL), suramin (100 μM), RB‐2 (100 μM), MRS2500 (1 μM), AR‐C118925XX (1 µM), NF157 (5 μM) or 5‐BDBD (10 μM) or inhibitors of transcription (actinomycin D, 1 μM in DMSO) or translation (cycloheximide, 10 μg/mL). Cells were pre‐incubated with these inhibitors at 37°C for 30 min. Stimulations were carried out for 18 h in a fresh medium. Media were then collected and centrifuged to remove detached cells and were analysed for the detection of human CXCL8/IL‐8 and CXCL10/IP‐10 using ELISA kits.

### 2.4. Quantitative Real‐Time PCR (RT‐qPCR)

HT‐29 cells at passage 3 were seeded into 24‐well plates at a density of 2 × 10^5^ cells per well in 1 mL of the medium. After 24 h of incubation, cells were pre‐treated with apyrase (2 U/mL) in fresh medium for 15 min, followed by stimulation with poly(I:C) at 1 μg/mL or 10 μg/mL (three wells per condition) for 18 h. Supernatants were collected, and cells were lysed with 0.25 mL of TRIzol reagent per well. Lysates from three wells were pooled for RNA extraction, which was performed using the TRIzol reagent according to the manufacturer’s instructions. Total RNA was quantified using the Quant‐iT RNA BR assay kit and a Qubit fluorometer. RNA extraction, cDNA synthesis, and quantitative analysis were carried out following previously published protocols [21].

RT‐qPCR was performed using 1 µL of cDNA, 5 µL of FastStart Universal SYBR Green Master (ROX), and 1 µL of gene‐specific primers (3 µM) in a 10 µL reaction volume on a MicroAmp optical 384‐well plate using an Applied Biosystems 7900HT Fast Real‐Time PCR system. The cycling conditions were as follows: 2 min at 50°C and 10 min at 95°C, followed by 40 cycles of 15 s at 95°C and 1 min at 60°C (or 57.5°C for P2X5 and P2X7), with a final dissociation step. For absolute quantification, standard curves were generated using serial dilutions from 10 to 10^9^ copies of the target amplicon, and results were expressed as copy/µL of cDNA. Negative controls included reactions with water as the template. For relative quantification, the 2^*–*ΔΔCt^ method was used, and expression levels were normalised to GAPDH. GAPDH was also used as a control for DNA quality in semi‐quantitative (2 min at 94°C, 30 cycles of 45 s at 94°C, 45 s at 60°C, 45 s at 72°C and a final 7 min at 72°C). The primer sequences used for semi‐quantitative PCR or RT‐qPCR are listed in Table 1.

**Table 1:** RT‐qPCR primers.

Gene	Forward primer	Reverse primer	Amplicon
*GAPDH*	GAGTCAACGGATTTGGTCGT	TTGATTTTGGAGGGATCTCG	238
*P2RX1*	GCTACGTGGTGCAAGAGTCA	GTAGTTGGTCCCGTTCTCCA	215
*P2RX2*	Qiagen, QT00493682	Qiagen, QT00493682	117
*P2RX3*	Qiagen, QT00026446	Qiagen, QT00026446	132
*P2RX4*	Qiagen, QT00049693	Qiagen, QT00049693	103
*P2RX5*	CTGGTCGTATGGGTGTTCCT	CTGGGCTGGAATGACGTAGT	159
*P2RX6*	Qiagen, QT00018949	Qiagen, QT00018949	124
*P2RX7*	AAGCTGTACCAGCGGAAAGA	GCTCTTGGCCTTCTGTTTTG	202
*P2RY1*	AAAACTAGCCCCCTGCAACT	GATCTGATGCCGGATGAACT	153
*P2RY2*	CCACCTGCCTTCTCACTAGC	TGGGAAATCTCAAGGACTGG	163
*P2RY4*	CACCCGCACCATTTACTACC	CCCCAGTGAGCAAGTAGAGC	163
*P2RY6*	AGCTGGGCATGGAGTTAAGA	GCTGACTGGGACCTCTCAAG	139
*P2RY11*	CCTCTACGCCAGCTCCTATG	CACTGCGGCCATGTAGAGTA	211
*P2RY12*	TTTGCCCGAATTCCTTACAC	ATTGGGGCACTTCAGCATAC	192
*P2RY13*	CCCCTGGTACACTTGGAAGA	TACAGAGGAGGGGGTGATTG	125
*P2RY14*	Qiagen, QT00199997	Qiagen, QT00199997	110
*CXCL8*	TCTGCAGCTCTGTGTGAAGGT	GCTTGAAGTTTCACTGGCATC	313
*CXCL10*	Qiagen, QT01003065	Qiagen, QT01003065	129

### 2.5. siRNA Transfection and Gene Expression

HT‐29 cells from passages 2–3 were resuspended at a concentration of 1 × 10^6^ cells in 100 μL of Amaxa Cell Line Nucleofector Kit R solution, containing 300 nM of *P2RY2* or a negative control (scrambled siRNA). The cell suspension was transferred to a nucleofector cuvette and transfected using the Nucleofector II device (Amaxa Biosystems) with programme W‐017 for high transfection efficiency. After nucleofection, cells were diluted in pre‐warmed complete medium and seeded into 24‐well plates at a density of 0.5 × 10^6^ cells per well in a final volume of 1 mL. Forty‐eight hours post‐transfection, cells were treated with poly(I:C) or appropriate controls. Five hours after the later stimulation, supernatants were collected, and cells were lysed with the TRIzol reagent for RNA extraction and subsequent RT‐qPCR, as described above.

### 2.6. Quantification of Extracellular ATP

HT‐29 cells were seeded at a density of 2 × 10^5^ cells per well in 24‐well plates and cultured for 24 h or 4 days. The culture medium was then replaced with ATP‐release buffer (1x HBSS, 1 mM MgCl_2_, 2.5 mM CaCl_2_, 5 mM Hepes, pH 7.4), and the cells were left untreated or treated with poly(I:C) (1 µg/mL) or LPS (0.1 µg/mL) for various time periods (2, 5, 15, and 30 min; 1, 4, 8, 16, and 24 h). At each time point, 200 µL of buffer was collected on ice and centrifuged at 3000 rpm for 1 min at 4°C to remove debris. ATP levels in the supernatant were measured using the luciferin/luciferase ATP Bioluminescence Assay Kit CLS II (Roche) according to the manufacturer’s instructions, and luminescence was recorded with a Varioskan LUX multimode microplate reader (Thermo Scientific, Waltham, MA, USA). ATP quantities were normalised to account for the total buffer volume per well, allowing direct comparison across time points and conditions.

### 2.7. Animals

All experiments were conducted in accordance with the guidelines of the Canadian Council on Animal Care, and protocols were approved by the Animal Care Committee of Université Laval. *P2yr2* ^*-/*-^ mice obtained from B. Robaye (Université Libre de Bruxelles, Belgium) [27, 28] were backcrossed 15 times with C57BL/6 mice from Charles River Laboratories (Pointe‐Claire, Quebec, Canada). *P2yr2* ^*-/*-^ mice as well as C57BL/6 mice (8–12 weeks old) were bred and maintained under specific pathogen‐free conditions.

### 2.8. IEC Isolation

Primary IECs were isolated from wild‐type (WT) and *P2ry2* ^-/-^ mice following the protocol described previously [8, 16]. The isolated crypts were resuspended and seeded in 500 μL of complete culture medium supplemented with growth factors in a 24‐well plate pre‐coated with type I collagen at a density of 1000 crypts per well. Cells were then incubated at 37°C, 5% CO_2_, with media replacement on day 2 (without Y‐27632) and cultured for 4 days until confluence to obtain a differentiated IEC monolayer.

### 2.9. IEC Stimulation and CXCL1/KC ELISA

IECs were stimulated in a growth factor‐free medium for 18 h with poly(I:C) at concentrations of 0.1, 1 and 10 μg/mL. After stimulation, cell culture medium was harvested on ice and centrifuged at 1500 rpm for 10 min at 4°C. The resulting supernatants were carefully collected and stored at −80°C until CXCL1/KC quantification by ELISA according to the manufacturer’s instructions (R&D Systems, Inc., Minneapolis, MN, USA).

### 2.10. Statistical Analysis

Data analysis was performed using GraphPad Prism Version 10.2.3. Ordinary one‐way ANOVA was used for comparisons involving more than two groups, and ordinary two‐way ANOVA was applied when evaluating multiple parameters across two or more groups. Dunnett’s post hoc test was used for multiple comparisons. *p*‐values < 0.05 were considered statistically significant ( ^∗^ *p*  < 0.05,  ^∗∗^ *p*  < 0.01,  ^∗∗∗^ *p*  < 0.001,  ^∗∗∗∗^ *p*  < 0.0001).

## 3. Results

### 3.1. HT‐29 Cells Release Chemokines Upon Stimulation With PAMPs

**Figure 1:**
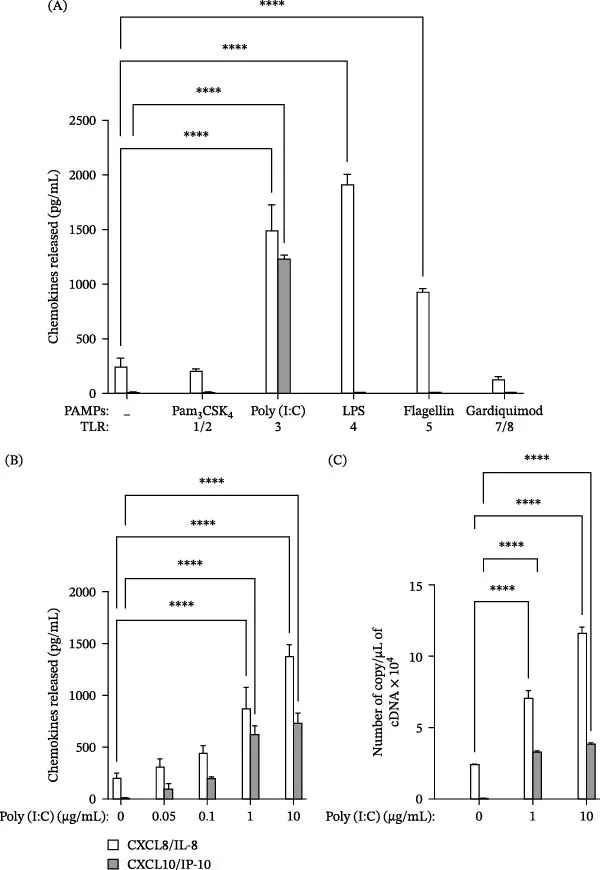
PAMPs induce chemokine release by HT‐29 cells. (A) The levels of CXCL8/IL‐8 and CXCL10/IP‐10 in the supernatant of HT‐29 cells treated with Pam_3_CSK_4_ (1 μg/mL), poly(I:C) (1 μg/mL), LPS (0.1 μg/mL), flagellin (1 μg/mL) or gardiquimod (1 μg/mL) for 18 h were determined by ELISA (*n* = 3). (B) The concentration‐dependent response of poly(I:C)‐induced CXCL8/IL‐8 and CXCL10/IP‐10 protein levels in these cells was assessed by ELISA (*n* = 3). (C) The concentration‐dependent response of poly(I:C)‐induced CXCL8/IL‐8 and CXCL10/IP‐10 mRNA expression in HT‐29 cells were assessed by RT‐qPCR using GAPDH as an internal control (*n* = 2). Data were analysed by two‐way ANOVA with multiple comparisons (one family per column; subfigures A–C)  ^∗∗∗∗^ *p*  < 0.0001 vs. controls, mean for all subfigures.

To explore the innate immune responsiveness of IECs to TLR agonists, HT‐29 cells were stimulated with various PAMPs recognised by TLRs. Supernatants were collected and assayed for CXCL8/IL‐8 and CXCL10/IP‐10 production by an ELISA. The following PAMPs were used: Pam_3_CSK_4_ (an agonist of TLR1/2), poly(I:C) (TLR3), LPS (TLR4), flagellin (TLR5) and gardiquimod (TLR7 and TLR8). As indicated in Figure 1A, the ligands of TLR3, TLR4 and TLR5 induced secretion of CXCL8/IL‐8 during the 18 h incubation period in HT‐29 cells. Since only poly(I:C) treatment induced the release of both CXCL8/IL‐8 and CXCL10/IP‐10, the effect of poly(I:C) was selected for further investigation.

To assess whether chemokine secretion was concentration‐dependent, HT‐29 cells were stimulated with increasing concentrations of poly(I:C). Protein levels of CXCL8/IL‐8 and CXCL10/IP‐10 in the culture supernatants were quantified by an ELISA. In parallel, gene expression of CXCL8/IL‐8 and CXCL10/IP‐10 in response to 1 and 10 μg/mL of the TLR3 ligand poly(I:C) was evaluated by RT‐qPCR. As shown in Figure 1B, CXCL8/IL‐8 and CXCL10/IP‐10 protein levels increased in a concentration‐dependent manner following poly(I:C) treatment of HT‐29 cells, as measured by the ELISA. The mRNA levels of both CXCL8 and CXCL10 exhibited a similar trend in response to poly(I:C) (Figure 1C).

### 3.2. Poly(I:C)‐Induced Secretion of Chemokines Is Unaffected by PKR Inhibition and Requires Endo‐Lysosomal Acidification

Viral dsRNA and its synthetic analogue poly(I:C) are also sensed by TLR‐independent pathways through the activation of the cytoplasmic RNA helicases retinoic acid‐inducible gene I (RIG‐I) and melanoma differentiation‐associated protein 5 (MDA5), as well as by RNA‐activated protein kinase (PKR), all of which are located in the cytosol. According to previous studies, poly(I:C) transfection is needed for efficient activation of these intra‐cytosolic dsRNA sensors as it enables direct delivery of poly(I:C) into the cytosol, bypassing the endosomal compartment [29, 30]. In contrast, when poly(I:C) is added to the culture medium (without transfection), it can activate surface TLR3 or can be internalised via endocytosis and reach endosomes, where it activates endosomal TLR3; especially, high‐molecular‐weight poly(I:C) (1.5–8 kb), which is known to induce robust CXCL10 release in HT‐29 cells by activating both surface and endo‐lysosomal TLR3 [6].

**Figure 2:**
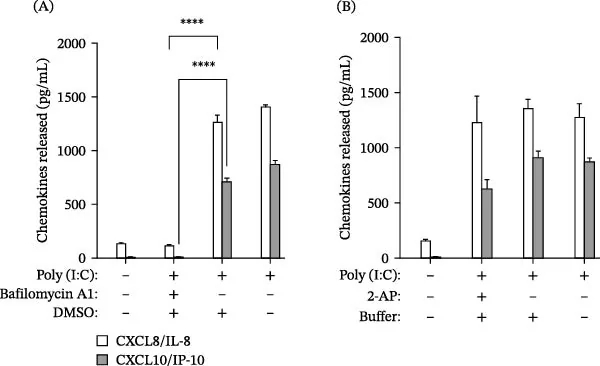
Bafilomycin A1 completely abolishes poly(I:C)‐induced chemokine release. (A) HT‐29 cells were left untreated or incubated with 50 ng/mL bafilomycin A1 or (B) 5 mM 2‐aminopurine (2‐AP) for 30 min prior to 18 h stimulation with poly(I:C) (1 μg/mL). Then, CXCL8/IL‐8 and CXCL10/IP‐10 in the supernatant were assessed by ELISA. The results are presented as mean ± SD of triplicates. All data were analysed using a two‐way ANOVA with a full model including the main effects of rows and columns, as well as their interaction. Tukey’s multiple‐comparisons test was used for post hoc analysis, with a single pooled variance.  ^∗∗∗∗^ *p*  < 0.0001.

In the present study, we used high‐molecular‐weight poly(I:C) without transfection to stimulate HT‐29 cells. Based on previous findings and because this form of poly(I:C) preferentially activates TLR3 when applied extracellularly, we hypothesised that the observed release of CXCL8/IL‐8 and CXCL10/IP‐10 was mainly due to TLR3 activation. To further evaluate the potential involvement of cytosolic dsRNA sensors such as PKR, we pre‐treated HT‐29 cells with 2‐aminopurine (a PKR inhibitor) and bafilomycin A1 (an inhibitor of vacuolar‐type H^+^‐ATPase) for 30 min prior to stimulation with poly(I:C). While bafilomycin A1 significantly reduced the secretion of both CXCL8/IL‐8 and CXCL10/IP‐10 (Figure 2A), 2‐aminopurine had no effect (Figure 2B). Altogether, these results show that poly(I:C)‐induced chemokine release in HT‐29 cells depends on endo‐lysosomal acidification that affects both endosomal and cell surface TLR3 activation. Notably, the cleaved form of TLR3 has been reported in the plasma membrane of HT‐29 cells [6]. Similar to the cathepsin inhibitor Z‐FA‐fmk, which blocks TLR3 cleavage and poly(I:C)‐induced cytokine secretion, bafilomycin A1 produced comparable effects [31], suggesting that it may impair cathepsin activity and consequently alter surface TLR3 signalling at these cell surfaces. This supports the idea that in our experimental conditions (i.e., without transfection), TLR3 is the primary sensor involved, while cytosolic receptors such as MDA5, RIG‐I or PKR are not significantly contributing to the observed effects.

### 3.3. Extracellular Nucleotides Are Essential for TLR3‐Induced CXCL8/IL‐8 and CXCL10 Release by HT‐29 Cells

**Figure 3:**
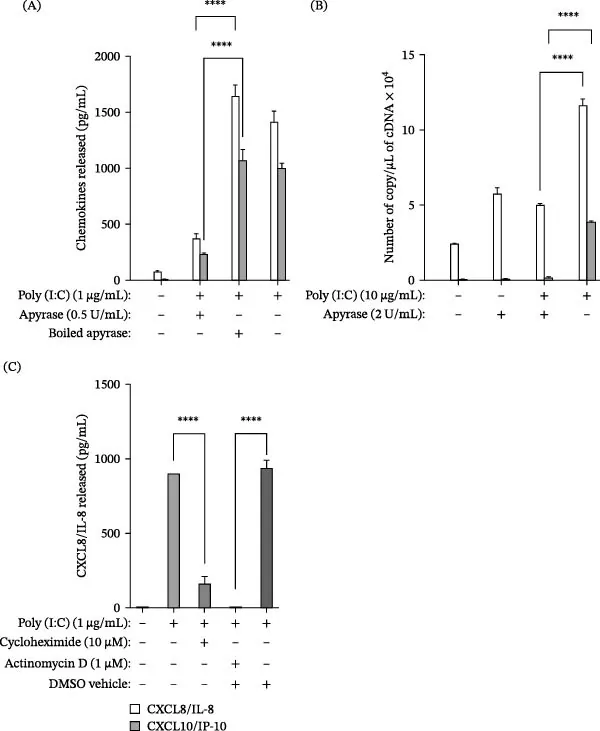
Extracellular nucleotides are required for poly(I:C)‐induced CXCL8/IL‐8 and CXCL10/IP‐10 production. (A) The CXCL8/IL‐8 and CXCL10/IP‐10 levels in the supernatant of HT‐29 cells stimulated for 18 h with poly(I:C) (1 μg/mL) alone or pre‐treated with apyrase (0.5 U/mL) or heat‐inactivated apyrase for 5 min before, were quantified by ELISA (mean ± SD of triplicates). (B) CXCL8/IL‐8 and CXCL10/IP‐10 mRNA levels in HT‐29 cells stimulated with poly(I:C) (10 μg/mL) for 18 h, with or without a 5‐min pre‐treatment with apyrase (2 U/mL), were assessed by RT‐qPCR (mean ± SD of two independent experiments). (C) HT‐29 cells were pre‐incubated for 30 min with the indicated concentrations of cycloheximide, actinomycin D or DMSO (0.1%), used as the vehicle control for actinomycin D before stimulation with poly(I:C) for 18 h. CXCL8/IL‐8 levels in the cell culture supernatants were then measured by ELISA (*n* = 3). Data from subfigures (A) and (B) were analysed using two‐way ANOVA with interaction, followed by Tukey’s post hoc test, while one‐way ANOVA was used for single‐factor comparisons in subfigure (C).  ^∗∗∗∗^ *p*  < 0.0001.

In previous reports, we have shown that TLR2‐ and TLR4‐induced CXCL8/IL‐8 release is associated with the concomitant activation of P2 receptors by extracellular nucleotides in monocytic cells and neutrophils [7, 11, 32]. To verify whether extracellular nucleotides were also involved in the effect observed with TLR3 activation here, we stimulated HT‐29 cells with poly(I:C) in the presence of apyrase, a nucleotide scavenger. Boiled apyrase (95°C, 10 min) was used as a control to confirm that the observed effects are due to the nucleotidase activity of apyrase. As shown in Figure 3A, treatment with an active apyrase at 0.5 U/mL abrogated the release of CXCL8/IL‐8 and CXCL10/IP‐10, whereas heat‐inactivated apyrase had no effect. In Figure 3B, apyrase at 2 U/mL significantly reduced the poly(I:C)‐induced mRNA expression levels of both CXCL8/IL‐8 and CXCL10/IP‐10. These results demonstrate that extracellular nucleotides play a pivotal role in mediating the release of CXCL8/IL‐8 and CXCL10/IP‐10 by HT‐29 cells in response to poly(I:C) stimulation.

To further assess whether the secreted CXCL8/IL‐8 was synthesised de novo following TLR3 stimulation, cells were treated with actinomycin D or cycloheximide to inhibit transcription or translation, respectively. As shown in Figure 3C, TLR3‐induced IL‐8 release was suppressed by both inhibitors, confirming that the IL‐8 released detected in the previous experiments originated from de novo synthesis rather than from pre‐existing intracellular stores.

### 3.4. Extracellular Nucleotide Signalling Regulates Poly(I:C)‐Induced Chemokine Release

**Figure 4:**
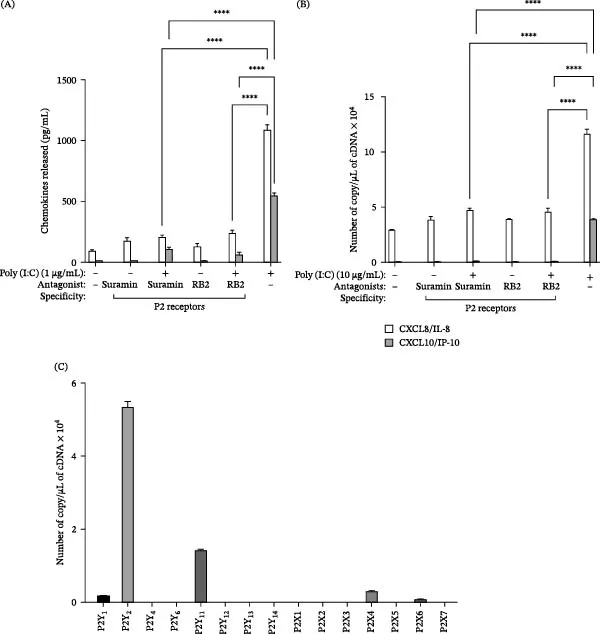
P2 general antagonists prevent poly(I:C)‐induced CXCL8/IL‐8 and CXCL10/IP‐10 expression and secretion by HT‐29 cells. (A) HT‐29 cells were stimulated with poly(I:C) (1 μg/mL) alone or in the presence of the broad‐spectrum P2 receptor antagonists suramin (100 μM) or Reactive Blue 2 (RB2; 100 μM), added 30 min prior to poly(I:C) stimulation. After 18 h, supernatants were collected, and chemokine levels were measured by ELISA. (B) HT‐29 cells were stimulated with poly(I:C) (10 μg/mL) alone or with suramin (100 μM) or RB2 (100 μM), added 30 min prior to poly(I:C) stimulation. Total RNA was extracted after 5 hours and the mRNA levels of CXCL8/IL‐8 and CXCL10/IP‐10 were quantified by absolute RT‐qPCR. (C) Basal mRNA expression levels of P2 receptor subtypes in unstimulated HT‐29 cells were analysed by RT‐qPCR. Data were analysed using two‐way ANOVA (subfigure A and B) including interaction, followed by Tukey’s post hoc test, or one‐way ANOVA (subfigure C).  ^∗∗∗∗^ *p*  < 0.0001. All results are mean of at least two independent experiments.

The apyrase‐mediated inhibition of TLR3‐induced CXCL8/IL‐8 and CXCL10/IP‐10 secretion by HT‐29 cells suggests that this response involved the activation of P2 receptors. To determine the role of P2 receptors in these two chemokines releases, we incubated HT‐29 cells with poly(I:C) in the presence of the general P2 receptor antagonists suramin and RB‐2. Both inhibitors markedly reduced poly(I:C)‐induced CXCL8/IL‐8 and CXCL10/IP‐10 secretion at the protein level (Figure 4A) as well as their mRNA expression levels (Figure 4B). To identify which P2Y receptor triggered CXCL8/IL‐8 and CXCL10/IP‐10 release in HT‐29 cells, we first analysed the repertoire of P2 receptors expressed in HT‐29 cells using RT‐qPCR. As seen in Figure 4C, HT‐29 cells expressed P2Y_1_, P2Y_2_, P2Y_11_ and P2X4 receptors. A weak signal was also detected for P2X6, which is generally considered non‐functional as a homomeric receptor in humans. P2X6 can form functional heteromeric receptors with P2X2; however, P2X2 expression was not detected under our experimental conditions.

### 3.5. Poly(I:C)‐Induced Chemokine Production by HT‐29 Cells Is Controlled by the P2Y_2_ Receptor

**Figure 5:**
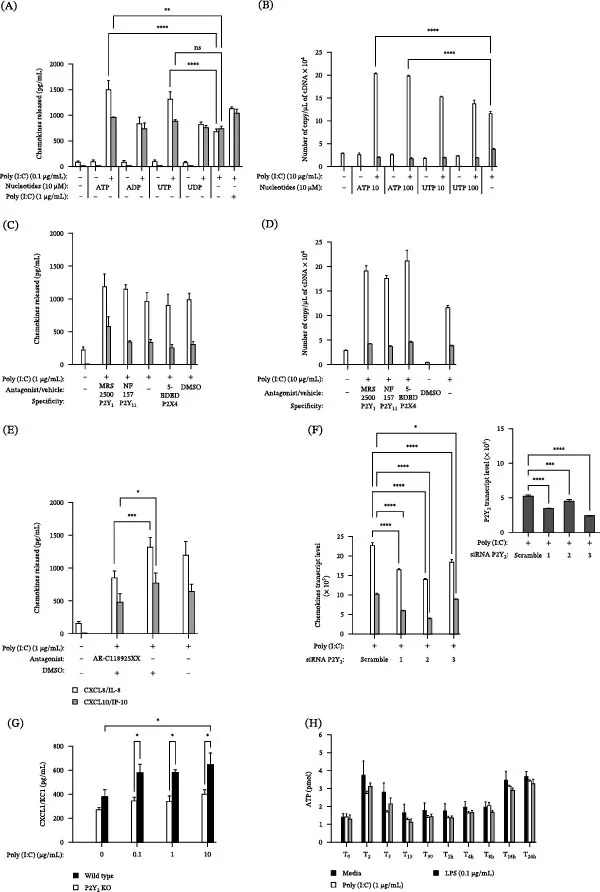
P2Y_2_ receptor activation is required for poly(I:C)‐mediated CXCL8/IL‐8 production in intestinal epithelial cells. (A) HT‐29 cells were stimulated with exogenous nucleotides (10 μM) either alone or in combination with suboptimal‐concentration poly(I:C) (0.1 μg/mL) for 18 h. CXCL8/IL‐8 levels in culture supernatants were quantified by ELISA (*n* = 3) (B) HT‐29 cells were treated with ATP or UTP (10 or 100 μM), either alone or in combination with poly(I:C) (10 μg/mL) for 5 hours. CXCL8/IL‐8 and CXCL10/IP‐10 mRNA expression levels were assessed by RT‐qPCR (*n* = 2). (C) HT‐29 cells were pre‐treated for 30 min with selective antagonists targeting P2Y_1_ (MRS2500, 1 μM), P2Y_11_ (NF157, 5 μM) or P2X4 (5‐BDBD, 10 μM), followed by stimulation with poly(I:C) (1 μg/mL) for 18 h. CXCL8/IL‐8 and CXCL10/IP‐10 levels in culture supernatants were measured by ELISA (*n* = 3). (D) HT‐29 cells were pre‐treated with the same P2 receptor antagonists for 30 min before stimulation with poly(I:C) 10 μg/mL for 5 h. CXCL8/IL‐8 and CXCL10/IP‐10 mRNA expression levels were analysed by RT‐qPCR (*n* = 2). (E) HT‐29 cells were pre‐treated for 30 min with selective P2Y_2_ antagonist AR‐C118925XX (10 μM) before stimulation with poly(I:C) 1 μg/mL for 18 h. CXCL8/IL‐8 and CXCL10/IP‐10 levels in culture supernatants were quantified by ELISA (*n* = 3). (F) HT‐29 cells were transfected with three independent P2Y_2_‐targeting siRNAs (1, 2 and 3) or a scramble siRNA. Forty‐eight hours post‐transfection, cells were stimulated with poly(I:C) (10 μg/mL) for 5 h. CXCL8/IL‐8 and CXCL10/IP‐10 mRNA expression levels were quantified by RT‐qPCR. Inset shows relative P2Y_2_ mRNA expression (after 48 h transfection) in siRNA‐treated cells, as assessed by RT‐qPCR (*n* = 3). (G) Primary intestinal epithelial cells were isolated from wild‐type and P2Y_2_‐knock‐out mice and stimulated with increasing concentrations of poly(I:C) for 18 h. CXCL1/KC levels in culture supernatants were quantified by ELISA (*n* = 2). (H) Extracellular ATP release by untreated HT‐29 cells or cells stimulated with poly(I:C) or LPS for the indicated times was measured using a luciferin–luciferase assay (*n* = 3). Data were analysed using one‐way or two‐way ANOVA, as appropriate. Two‐way ANOVA was used to evaluate the effects of two independent variables and their interaction, followed by Tukey’s or Dunnett’s multiple‐comparisons test with a single pooled variance. One‐way ANOVA was used for single‐factor comparisons ( ^∗^ *p*  < 0.05,  ^∗∗^ *p*  < 0.01,  ^∗∗∗^ *p*  < 0.001,  ^∗∗∗∗^ *p*  < 0.0001).

To discriminate between these receptor subtypes in CXCL8/IL‐8 and CXCL10/IP‐10 release assays, HT‐29 cells were stimulated with exogenous nucleotides alone or in the presence of a suboptimal concentration of poly(I:C). The results show that nucleotides alone failed to elicit detectable release of CXCL8/IL‐8 and CXCL10/IP‐10. However, in the presence of low‐dose poly(I:C), both ATP and UTP, agonists of the P2Y_2_ receptor, significantly enhanced CXCL8/IL‐8 release (Figure 5A). ATP also significantly increased CXCL10/IP‐10 secretion under the same conditions. In these conditions, UTP had no effect on the IP‐10 release. At the mRNA level, co‐stimulation with 10 μg/mL poly(I:C) and either 10 or 100 μM ATP or UTP significantly upregulated CXCL8/IL‐8 mRNA expression but not CXCL10/IP‐10 (Figure 5B).

HT‐29 cells were then stimulated with poly(I:C) in the presence of MRS2500 (1 μM), AR‐C18925XX (1 and 10 μM), NF157 (5 μM) and 5‐BDBD (10 μM), selective antagonists for P2Y_1_, P2Y_2_, P2Y_11_ and P2X4 receptors, respectively, which together represent the complete set of nucleotide receptors expressed in HT‐29 cells. In these assays, selective antagonists of P2Y_1_, P2Y_11_ and P2X4 had no significant effect on the release (Figure 5C) or mRNA expression (Figure 5D) of either chemokine in HT‐29 cells, in contrast to the inhibitory effects observed above with apyrase, suramin, and RB‐2 (Figures 3A,B and 4A,B). Only AR‐C118925XX significantly reduced poly(I:C)‐induced chemokines release by HT‐29 cells, suggesting the involvement of the P2Y_2_ receptor in this process (Figure 5E). The knockdown of the receptor with specific siRNAs also led to a significant reduction of CXCL8/IL‐8 and CXCL10/IP‐10 mRNA (Figure 5F). Altogether, these results show that the TLR3‐induced CXCL8/IL‐8 and CXCL10/IP‐10 response require the activation of P2Y_2_ receptors in HT‐29 cells.

In agreement, primary IECs from P2Y_2_ knock‐out (KO) mice produce significantly less CXCL1/KC in response to poly(I:C) stimulation compared to their WT counterparts (Figure 5G). Other cytokines, including CCL2/MCP‐1, CXCL2/MIP‐2, and murine CXCL10/IP‐10, were also tested (data not shown) but did not exhibit a significant increase following poly(I:C) stimulation. We therefore focused our analysis on CXCL1, which showed a clearer and readily measurable response. Note that we used KC (CXCL1), one of the functional murine counterparts of human IL‐8, since IL‐8 is not expressed in mice. Although KC is not genetically identical to IL‐8, it fulfils similar roles in neutrophil recruitment and inflammatory signalling [33]. Thus, these findings extend the association between P2Y_2_ and poly(I:C)‐induced chemokine production to primary mouse IECs.

To determine whether HT‐29 cells release ATP upon stimulation with poly(I:C) or LPS, we performed luciferin–luciferase assays in 24‐well plates. Because medium replacement and buffer handling may induce transient mechanical/shear stress, part of the early ATP signal observed in unstimulated cells may reflect handling‐associated basal ATP release rather than ligand‐induced ATP secretion. However, since both poly(I:C)‐treated cells and buffer‐only controls were subjected to the same handling procedures, any difference between these conditions would reflect a ligand‐specific effect rather than shear stress. The results showed that neither poly(I:C) at 1 μg/mL nor LPS at 0.1 μg/mL induced an increase in ATP release in HT‐29 cells (Figure 5H). Nevertheless, it is noteworthy that these cells exhibited a constitutive ATP release of 1–2 pmol per well, detectable within a short incubation period (2–5 min). This endogenous ATP release appears sufficient to activate P2Y receptors and is essential for the observed chemokine production. Collectively, these results demonstrate that the TLR3‐induced chemokine response in HT‐29 cells requires concomitant activation of P2Y_2_ receptors.

We next asked whether extracellular nucleotides and the P2Y_2_ receptor also contributed to poly(I:C)‐induced chemokine production in an inflammatory‐like context. HT‐29 cells were therefore pre‐treated with TNF and IFN‐γ in a serum‐free medium before poly(I:C) stimulation, to mimic an inflamed epithelial environment while minimising potential interference from serum‐derived factors [34]. Cell viability was monitored to ensure that serum starvation did not affect the interpretation of the results [35]. Although these conditions may not be ideal for assessing TLR‐specific responses because TNF and IFN‐γ pre‐treatment induced chemokine release by themselves, they remain relevant to evaluate nucleotide receptor involvement in an inflammatory epithelial context.

Because inflammatory cytokines may alter nucleotide receptor expression, we have also evaluated the expression of the most important P2 receptors found to be expressed in HT‐29 cells under normal conditions. Based on the basal expression profile shown in Figure 4C and considering the established roles of purinergic receptors in intestinal epithelial physiology and inflammation, we selected P2Y_1_, P2Y_2_, P2Y_6_, P2Y_11_, P2X4 and P2X7 for further analysis. P2Y_6_ was included because UDP‐P2Y_6_ signalling contributes to inflammatory chemokine production and epithelial restitution in the intestine [8, 15, 36], whereas P2X7 was examined because extracellular ATP–P2X7 signalling has been implicated in intestinal epithelial inflammation, barrier dysfunction and cell death [37, 38].

**Figure 6:**
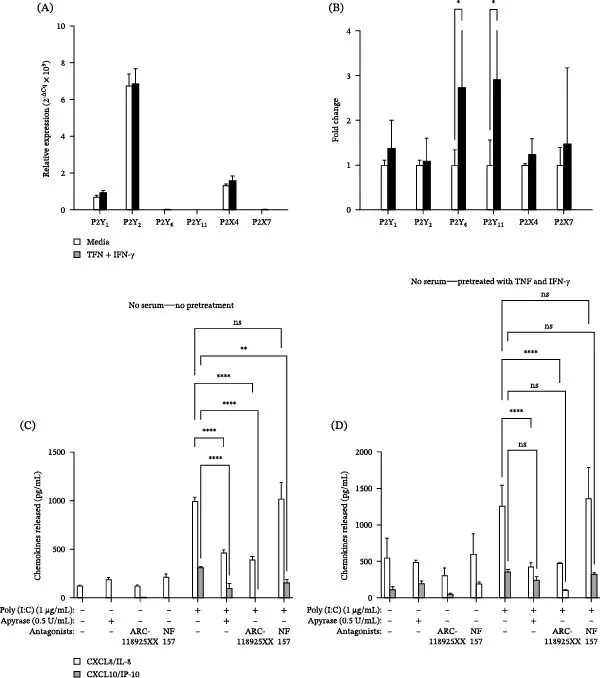
P2Y_2_ regulates poly(I:C)‐induced chemokine secretion in resting and TNF/IFN‐γ‐pre‐treated HT‐29 cells. (A) Relative mRNA expression of several P2 receptors, as indicated, in HT‐29 cells cultured in serum‐free medium alone or pre‐treated for 6 h with TNF + IFN‐γ (*n* = 3). (B) Fold‐change analysis of nucleotide receptor expression after TNF + IFN‐γ pre‐treatment compared with serum‐free medium alone (*n* = 3). (C) HT‐29 cells were stimulated with poly(I:C) under resting serum‐free conditions in the presence or absence of apyrase or selective P2Y_2_ and P2Y_11_ antagonists, and chemokine release was assessed by ELISA. (D) HT‐29 cells were pre‐treated for 6 h with TNF + IFN‐γ under serum‐free conditions. The medium was then removed, and cells were stimulated with poly(I:C) under serum‐free conditions in the presence or absence of apyrase or selective P2Y_2_ and P2Y_11_ antagonists. Chemokine release was assessed by ELISA. Two‐way ANOVA was used to evaluate the effects of two independent variables and their interaction, followed by Tukey’s or Dunnett’s multiple‐comparisons test with a single pooled variance ( ^∗^ *p* < 0.05,  ^∗∗^ *p*  < 0.01,  ^∗∗∗∗^ *p*  < 0.0001).

Consistent with the basal expression profile shown in Figure 4C, P2Y_2_ was the most highly expressed receptor under both control and inflammatory conditions, followed by P2X4 and P2Y_1_ (Figure 6A). TNF and IFN‐γ pre‐treatment did not significantly alter the P2Y_2_ expression. However, fold‐change analysis revealed significant increases in P2Y_6_ and P2Y_11_ expression, whereas the changes observed for P2Y_1_, P2Y_2_, P2X4 and P2X7 were not statistically significant (Figure 6B). Despite their induction, P2Y_6_ and P2Y_11_ remained expressed at substantially very low levels when compared with those of P2Y_2_. Thus, inflammatory cytokine pre‐treatment affected only very modestly the expression of P2 receptors without changing the predominance of any of them in HT‐29 cells.

Under serum‐free conditions, in resting HT‐29 cells, apyrase, used as a nucleotide‐scavenging enzyme, markedly reduced the release of both chemokines. A comparable inhibition was observed with the P2Y_2_ antagonist ARC‐118925XX, further supporting the role for P2Y_2_ in the nucleotide‐dependent amplification of CXCL8/IL‐8 and CXCL10/IP‐10 secretion (Figure 6C). By contrast, P2Y_11_ antagonist NF157 mainly affected CXCL10/IP‐10 secretion, while CXCL8/IL‐8 was not significantly altered under these resting serum‐free conditions (Figure 6C). In TNF + IFN‐γ‐pre‐treated cells, apyrase and ARC‐118925XX showed a similar inhibitory profile, particularly on CXCL8/IL‐8 secretion, whereas NF157 did not significantly reduce chemokine release (Figure 6D). Overall, these data indicate that P2Y_2_ consistently contributes to poly(I:C)‐induced chemokine secretion in both resting and inflammatory‐like conditions, whereas the expression and contribution of P2Y_11_ appear to be more variable and context‐dependent (data not shown).

## 4. Discussion

The present study demonstrates that stimulation of TLR3 in HT‐29 cells induces the production and release of the chemokines CXCL8/IL‐8 and CXCL10/IP‐10 in a concentration‐dependent manner and that this response critically depends on the concomitant activation of nucleotide P2 receptors. Specifically, upon exposure to the TLR3 ligand poly(I:C), HT‐29 colon adenocarcinoma cells exhibited robust expression and secretion of both chemokines. Interestingly, this response was abolished by the enzymatic degradation of extracellular nucleotides using apyrase (an enzyme that hydrolyses P2 receptor ligands) and by broad‐spectrum P2 receptor antagonists, as well as by selective P2Y_2_ antagonists and siRNA‐mediated knockdown of the receptor. These findings indicate that TLR3 activation alone is insufficient to elicit CXCL8/IL‐8 and CXCL10/IP‐10 production and that co‐activation of extracellular nucleotide receptors is essential to trigger this response. Importantly, our data indicate that HT‐29 cells released constitutively extracellular ATP and that this release was enhanced neither by TLR3 activation nor by LPS activation. Therefore, this constitutively released extracellular ATP appears to provide a basal nucleotide signal, which allows poly(I:C)‐induced chemokine production. By contrast, poly(I:C) has been reported to trigger ATP release in astrocytes and retinal pigmented epithelial cells [39]. It is therefore plausible that, in vivo, TLR3 activation may induce nucleotide release in certain cell types, which could, in turn, modulate the responses of neighbouring cells, including their TLR3‐mediated responses.

The analysis of P2 receptor expression profiles in HT‐29 cells revealed a predominant expression of P2Y_2_ mRNA (Figure 4C), consistent with previous findings [20], followed by P2Y_11_, P2X4 and P2Y_1_. This expression and functional pattern suggest that P2Y_2_ may play a central role in nucleotide‐mediated signalling in this cellular context. In the present study, we also show that IECs from P2Y_2_ KO mice produced significantly less CXCL1/KC compared to their WT counterparts upon poly(I:C) stimulation (Figure 5G), extending the association between P2Y_2_ and poly(I:C)‐induced chemokine production to primary mouse IECs.

Consistent with the observations made in this report, we previously demonstrated that activation of P2Y_2_ and/or P2Y_6_ receptors is required for TLR1/2‐induced CXCL8/IL‐8 release in monocytes and neutrophils [7, 12, 32]. We also showed that purine‐metabolising ectoenzymes at the surface of HT‐29 cells, particularly NTPDase2 and adenylate kinase (ADK), affected CXCL8/IL‐8 production in HT‐29 cells, suggesting that extracellular nucleotide signalling was involved in these effects [21].

In line with these findings, other studies have demonstrated a role of the P2Y_2_ receptor in cytokine secretion across different cell types. ATP was shown to induce a dose‐dependent release of IL‐8 in air‐liquid interface‐cultured human oesophageal epithelial cells through a P2Y_2_ receptor activation, which in turn triggered the extracellular signal‐regulated kinase (ERK) pathway [40]. More recently, TLR3‐induced interferon secretion in airway epithelial cells was reported to be suppressed by P2Y_2_ receptor activation through a mechanism involving Gq/PKC signalling and transcriptional inhibition of the *IFNβ1* gene [41]. The P2Y_2_ receptor emerges as a key mediator in the regulation of inflammation through its multifaceted role in epithelial cell signalling. In addition, during inflammatory conditions such as inflammatory bowel disease, P2Y_2_ expression is markedly upregulated in IECs, a process tightly regulated by pro‐inflammatory transcription factors including NF‐κB, p65 and C/EBPβ, which synergistically enhance P2Y_2_ gene transcription [42, 43]. Additionally, P2Y_2_ stimulation promotes the expression of ICAM‐1 on the apical surface of IECs, facilitating the adhesion and transmigration of monocyte‐derived macrophages and, subsequently, neutrophils, an event that may exacerbate mucosal inflammation [17]. Furthermore, P2Y_2_ activation contributes to the expression of COX‐2 and the release of prostaglandin *E*_2_, further linking this receptor to both inflammatory amplification and epithelial restitution processes [42]. These findings highlight P2Y_2_ as a central integrator of signalling and inflammatory cytokine production in the gut epithelium, with implications for both the pathogenesis and resolution of intestinal inflammatory disorders.

## 5. Conclusion

The results of the present study support the conclusion that TLR3 stimulation with poly(I:C) requires nucleotide signalling for the production of CXCL8/IL‐8 and CXCL10/IP‐10 in human HT‐29 cells and CXCL1/KC in mouse primary IECs. This process involves ATP‐ and UTP‐activated P2Y_2_. These regulatory mechanisms of chemokine secretion in IECs may play a role in the pathogenesis of inflammatory bowel disease and related inflammatory conditions. These data, consistent with previous studies from our research team involving TLR2 and TLR4, suggest that extracellular nucleotides provide an essential co‐stimulatory signal for robust innate inflammatory responses. In the absence of nucleotide signalling, TLR activation alone appears insufficient to induce substantial cytokine production, highlighting the essential contribution of nucleotide signalling in orchestrating innate immune activation. In perspective, further studies are needed to decipher the downstream signalling crosstalk between PAMP‐activated TLR pathways and extracellular nucleotide/P2 receptor signalling in the regulation of chemokine production. It will be important to determine how TLR3‐dependent pathways, such as NF‐κB and IRF signalling, integrate with P2Y_2_‐mediated signals, including calcium mobilisation and MAPK activation, to amplify CXCL8/IL‐8 and CXCL10/IP‐10 expression. Future work should also clarify whether this cooperation occurs in primary IECs and in in vivo inflammatory models, where extracellular nucleotides may originate from multiple cellular sources within the intestinal microenvironment. Such studies would provide a better understanding of how danger‐ and pathogen‐associated signals cooperate to shape epithelial inflammatory responses and may help identify new therapeutic targets.

## Author Contributions

Abdoul Karim Ouattara participated in the study conceptualisation, carried out most of the experiments, analysed the data and draughted the manuscript. Fariborz Bahrami contributed to the study conceptualisation and experiments. Filip Kukulski, Radu Adrian Turcitu, Joanna Lecka, Nicolly Espindola Gelsleichter and Tais Vidal assisted with some experiments. Julie Pelletier was responsible for colony maintenance and helped with a few experiments. Jean Sévigny conceived the original idea, secured funding, supervised and administered the project and revised the manuscript.

## Funding

This study was supported by the Canadian Institutes of Health Research (Grants PJT ‐ 156205 and MOP‐93683) and the Fonds de recherche du Québec (Reference 30641).

## Disclosure

All authors have read the manuscript and approved the final version for publication.

## Conflicts of Interest

The authors declare no conflicts of interest.

## Data Availability

The data that support the findings of this study are available from the corresponding author upon reasonable request.
